# Falsely normalized ankle-brachial index despite the presence of lower-extremity peripheral artery disease: two case reports 

**DOI:** 10.1186/s13256-021-03155-z

**Published:** 2021-12-17

**Authors:** Tatsuya Maruhashi, Shogo Matsui, Farina Mohamad Yusoff, Shinji Kishimoto, Masato Kajikawa, Yukihito Higashi

**Affiliations:** 1 Department of Cardiovascular Regeneration and Medicine, Research Institute for Radiation Biology and Medicine (RIRBM), Hiroshima University, 1-2-3 Kasumi, Minami-ku, Hiroshima, 734-8553 Japan; 2 Department of Cardiovascular Medicine, Hiroshima University Graduate School of Biomedical Sciences, Hiroshima University, Hiroshima, Japan; 3 Division of Regeneration and Medicine, Hiroshima University Hospital, Hiroshima, Japan

**Keywords:** Ankle-brachial index, Peripheral artery disease, Infrapopliteal artery, Upstroke time, Diabetes mellitus, Atherosclerosis

## Abstract

**Background:**

The ankle-brachial index measurement is used for screening and diagnosis of lower-extremity peripheral artery disease and cardiovascular risk assessment. However, the value is occasionally unreliable since the oscillometric ankle-brachial index can be elevated and falsely normalized despite the presence of lower-extremity peripheral artery disease because of the incompressibility of infrapopliteal arteries at the ankle, potentially leading to a missed diagnosis of lower-extremity peripheral artery disease or underestimation of cardiovascular risk.

**Case presentation:**

We report two cases of lower extremity peripheral artery disease with normal ankle-brachial index (a 76-year-old Asian man and a 66-year-old Asian man). In both cases, the ankle-brachial index was within the normal range (1.00–1.40) despite the presence of lower-extremity peripheral artery disease, whereas upstroke time at the ankle calculated from the pulse volume waveform simultaneously obtained by plethysmography during the ankle-brachial index measurement was prolonged (≥ 180 milliseconds). Diagnostic imaging tests revealed the presence of occlusive arterial disease in the lower extremity and severe calcification of infrapopliteal arteries.

**Conclusions:**

In both cases, the oscillometric ankle-brachial index might have been falsely normalized despite the presence of lower-extremity peripheral artery disease because of calcified incompressible infrapopliteal arteries. Sole reliance on the ankle-brachial index value may lead to a missed diagnosis of lower-extremity peripheral artery disease or underestimation of cardiovascular risk. Upstroke time at the ankle was helpful for suspecting the presence of lower-extremity peripheral artery disease in both patients with normal ankle-brachial index. In addition to history-taking and vascular examination, upstroke time at the ankle should be carefully checked for accurate diagnosis of peripheral artery disease and cardiovascular risk assessment in patients with normal ankle-brachial index.

## Background

Ankle-brachial index (ABI) measurement has been widely used for screening and diagnosis of lower-extremity peripheral artery disease (PAD) and cardiovascular risk assessment. However, the results are occasionally unreliable for lower-extremity PAD screening and cardiovascular risk assessment since the oscillometric ABI value is elevated and falsely normalized despite the presence of occlusive arterial disease in the lower extremity in patients with incompressible infrapopliteal arteries, potentially leading to a missed diagnosis of PAD or underestimation of cardiovascular risk [1]. Incompressibility of infrapopliteal arteries is more common in elderly patients with diabetes mellitus and/or advanced chronic kidney disease. In these patients, a comprehensive approach that includes history-taking, physical examination, and other physiological tests is necessary to prevent a missed diagnosis of PAD or underestimation of cardiovascular risk.

We present here two cases of type 2 diabetes mellitus with normal ABI despite the presence of lower-extremity PAD, probably due to incompressible infrapopliteal arteries. In both cases, upstroke time calculated from the pulse volume waveform recording at the ankle was helpful for suspecting the presence of lower-extremity PAD.

## Case presentation

### Case 1

**Fig. 1:**
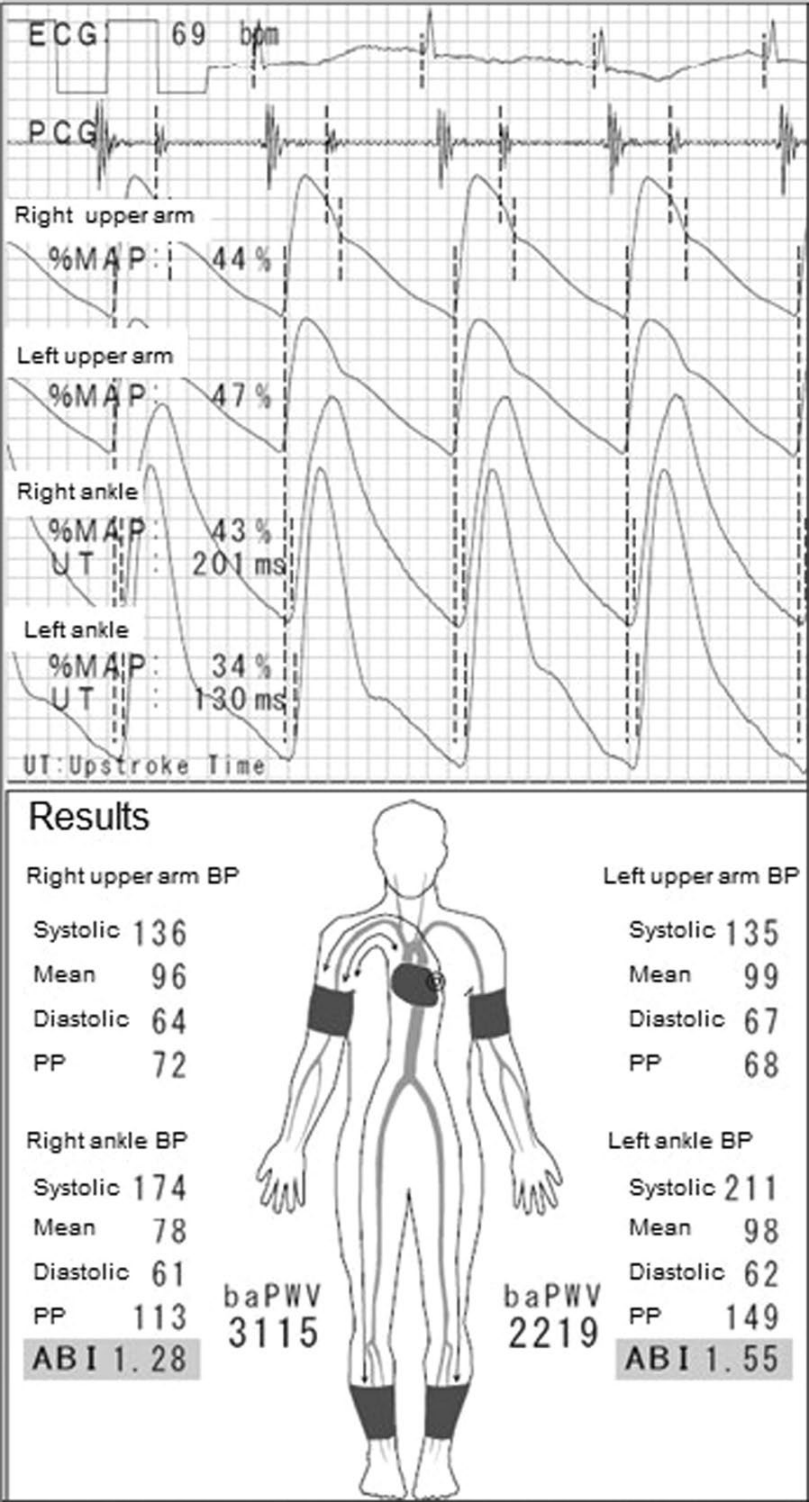
Ankle-brachial index measurement by volume plethysmography in patient 1. *ABI* ankle-brachial index, *UT* upstroke time, *BP* blood pressure, *PP* pulse pressure

**Fig. 2:**
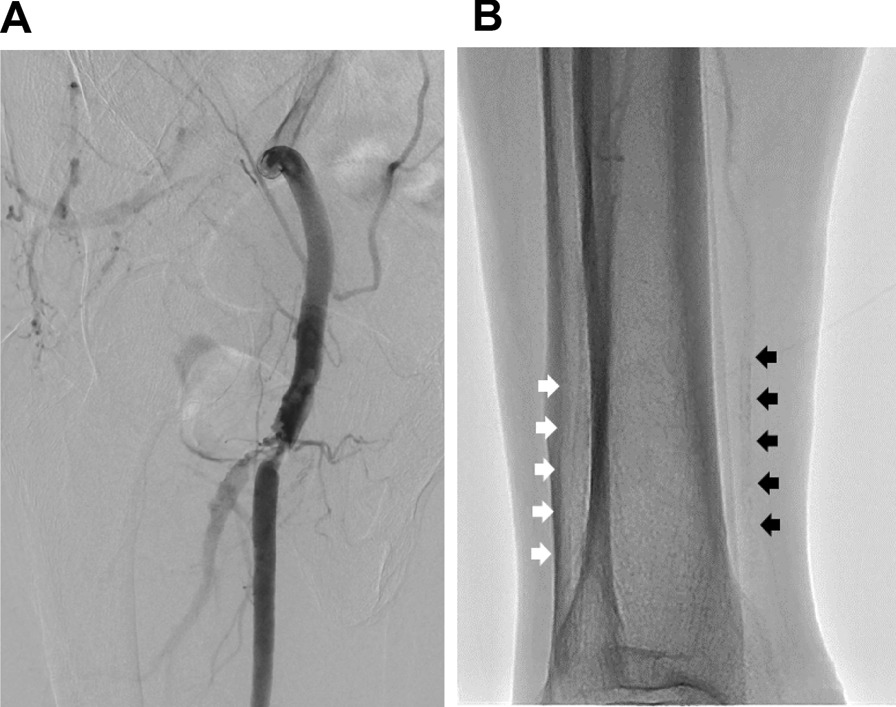
**A** Digital subtraction angiography in the right superficial femoral artery showing a severe stenotic lesion at the origin of the right superficial femoral artery. **B** Fluoroscopic image of the right lower leg showing severe calcification of the anterior tibial artery (white arrows) and posterior tibial artery (black arrows)

**Fig. 3:**
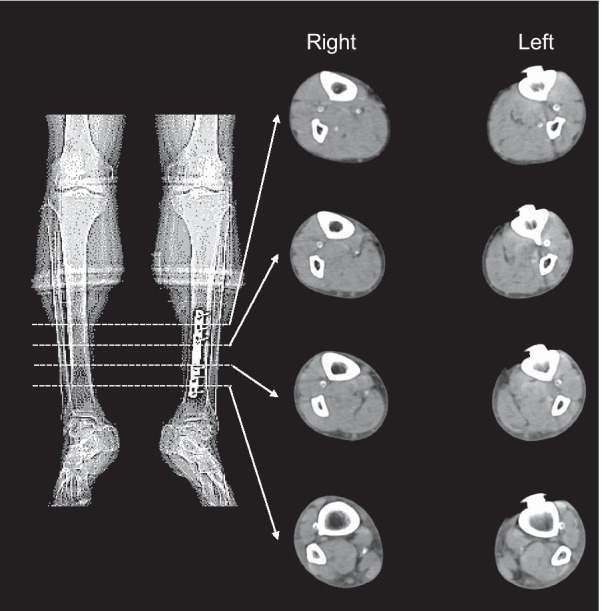
Computed tomography of the lower legs. Axial images of plain computed tomography showed severe calcification of infrapopliteal arteries in the bilateral lower legs

**Fig. 4:**
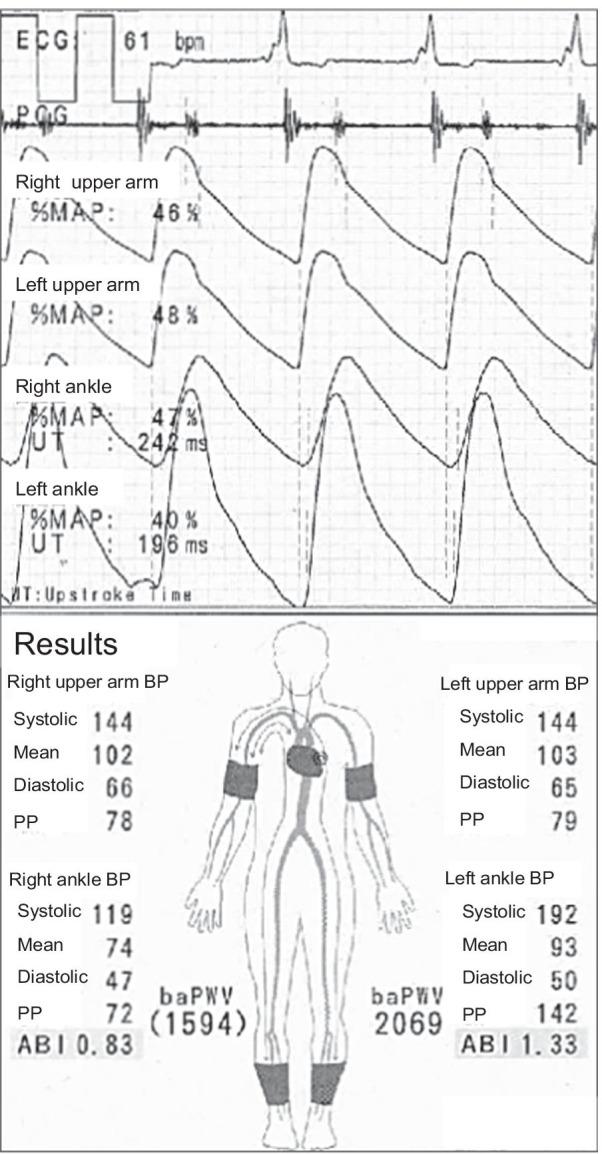
Ankle-brachial index measurement by volume plethysmography 5 years after the initial assessment in patient 1. *ABI* ankle-brachial index, *UT* upstroke time, *BP* blood pressure, *PP* pulse pressure

A 76-year-old Asian man with a 55-year history of type 2 diabetes mellitus was referred to our hospital for right intermittent claudication of 3 years. He had diabetic retinopathy and had been treated with insulin for 3 years. Vascular examination revealed right femoral bruit and diminished right popliteal pulse, suggesting the presence of a right femoral lesion. However, the right ABI measured by a volume plethysmograph (Form PWV/ABI; Omron Health Care Co., Kyoto, Japan) was 1.28, within the normal range (1.00–1.40) (Fig. 1). On the other hand, the left ABI was elevated to 1.55 (> 1.40), suggesting that the infrapopliteal arteries at the left ankle were incompressible. Upstroke time, the transit time from the nadir to the peak of the pulse volume waveform recording, of the right ankle was prolonged to 201 milliseconds (≥ 180 milliseconds), whereas upstroke time of the left ankle was within the normal range (130 milliseconds) (Fig. 1). These findings suggest that the right ABI was falsely normalized due to incompressible infrapopliteal arteries at the right ankle despite the presence of PAD in the right extremity. Indeed, intra-arterial angiography revealed the presence of a severe stenotic lesion at the origin of the right superficial femoral artery (SFA) (Fig. 2A), whereas there was no occlusive arterial disease in the left extremity. Fluoroscopic images showed severe calcification of infrapopliteal arteries at the right ankle (Fig. 2B). Computed tomography also revealed severe calcification of the bilateral infrapopliteal arteries (Fig. 3). Revascularization was not performed, and supervised exercise training was initiated. After completion of the 6-month supervised exercise training program, he continued to spend 2 hours a day walking. Five years from the initial assessment, the right ABI had decreased to 0.83, with upstroke time at the right ankle of 242 milliseconds, indicating the progression of the right lower extremity PAD (Fig. 4). However, there was no deterioration in his symptoms, and he had no difficulty in performing daily activities.

### Case 2

**Fig. 5:**
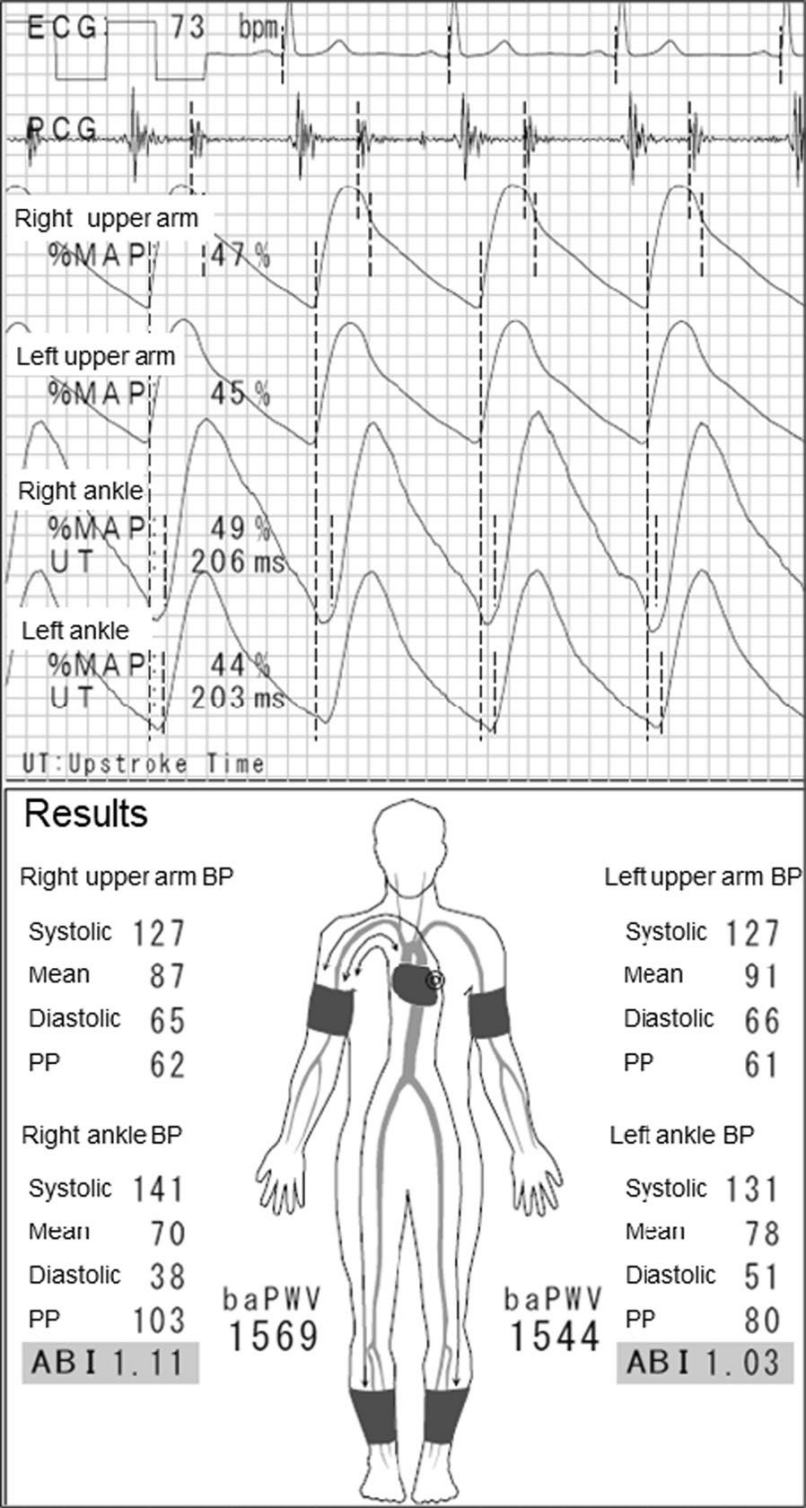
Ankle-brachial index measurement by a volume plethysmography in patient 2. *ABI* ankle-brachial index, *UT* upstroke time, *BP* blood pressure, *PP* pulse pressure

**Fig. 6:**
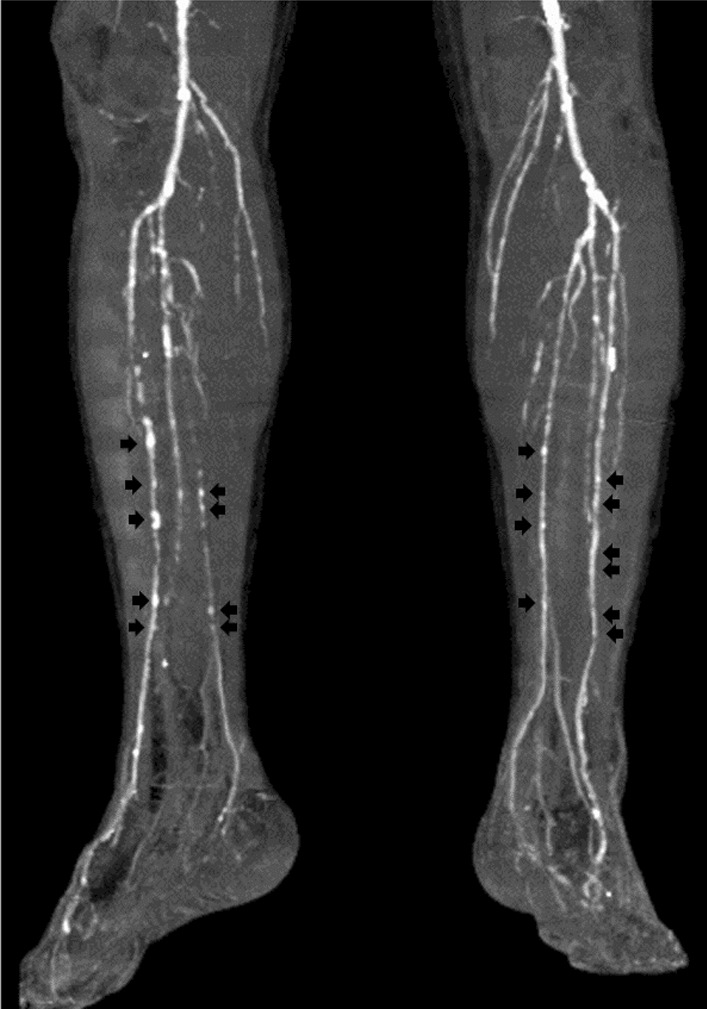
Computed tomography of the lower legs showing spotty calcification in the bilateral infrapopliteal arteries (black arrows)

**Fig. 7:**
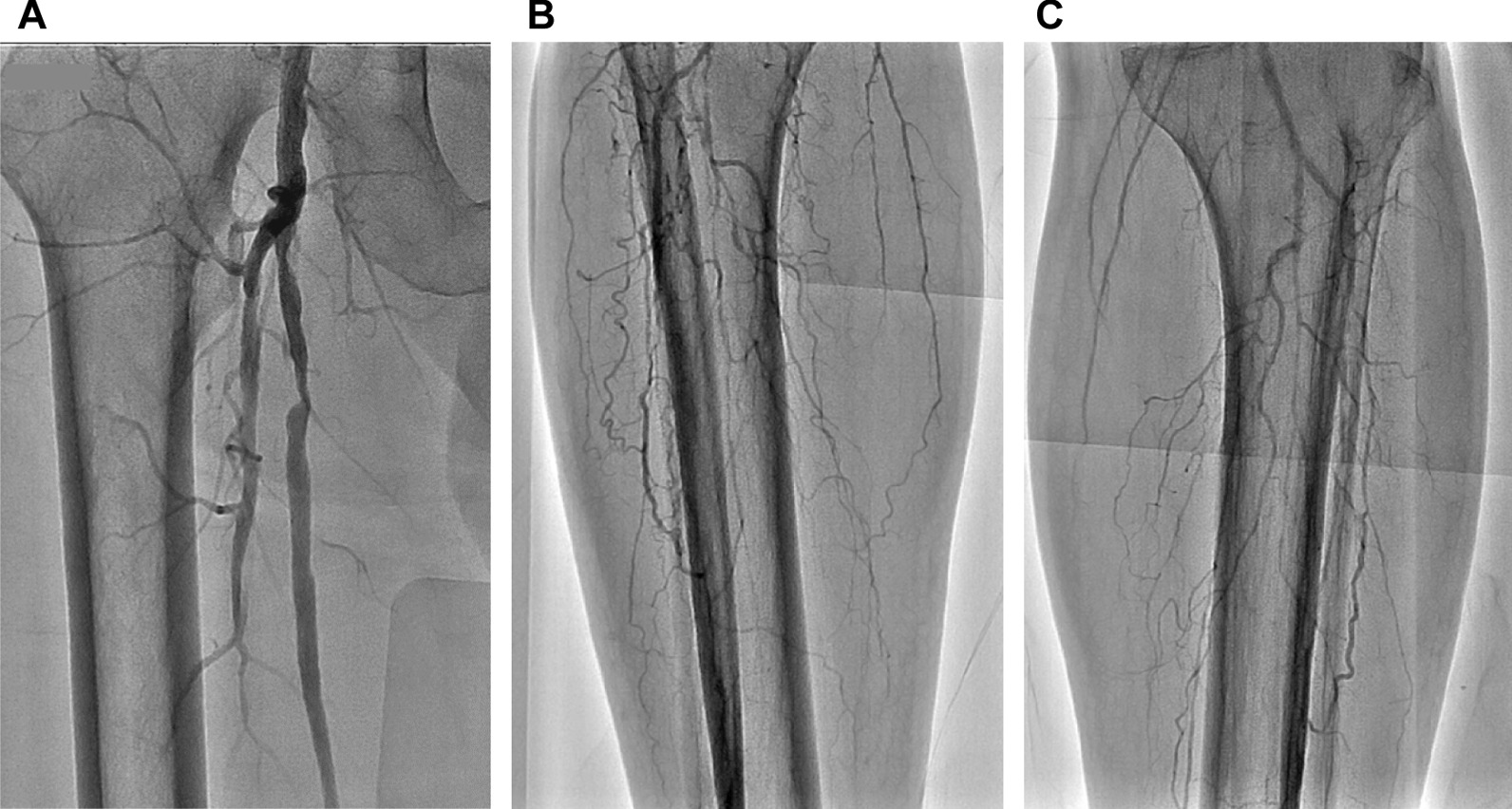
Intra-arterial angiography of the lower extremities showing severe stenotic lesions in the right superficial femoral artery (**A**), occlusive lesions in infrapopliteal arteries in the right lower leg (**B**) and left lower leg (**C**)

A 66-year-old Asian man with a 28-year history of type 2 diabetes mellitus was referred to our hospital for bilateral intermittent claudication of 6 months and exertional dyspnea. He had diabetic retinopathy and was being treated with insulin. He was an ex-smoker with a history of 40 pack-years. Vascular examination revealed right femoral bruit, diminished right popliteal pulse, and absent right and left dorsalis pedis pulses, suggesting the presence of right femoral and bilateral infrapopliteal lesions. However, both the right ABI (1.11) and left ABI (1.03) were within the normal range (Fig. 5). However, upstroke times were prolonged to 206 and 203 milliseconds (≥ 180 milliseconds) in the right and left ankles, respectively (Fig. 5), suggesting the presence of bilateral lower-extremity PAD. The right ABI decreased from 1.11 to 0.48 and the left ABI decreased from 1.03 to 0.67 after exercise. Computed tomography angiography revealed the presence of right SFA stenotic lesions, bilateral infrapopliteal occlusive lesions, and spotty calcification in bilateral infrapopliteal arteries (Fig. 6). Intra-arterial angiography revealed the presence of right SFA stenotic lesions and bilateral infrapopliteal occlusive lesions (Fig. 7A–C). Coronary angiography revealed severe stenotic lesions in the right coronary artery and the left anterior descending coronary artery (LAD). After percutaneous coronary intervention for the LAD stenosis, supervised exercise training was initiated.

## Discussion and conclusions

We describe here two patients with type 2 diabetes mellitus who had normal ABI despite the presence of lower-extremity PAD, probably because of incompressible infrapopliteal arteries at the ankle.

Measurement of the resting ABI is recommended as a first-line noninvasive test for screening and diagnosis of lower-extremity PAD [2, 3]. However, the sensitivity of ABI for diagnosis of lower-extremity PAD is poorer in patients with diabetes mellitus and/or advanced chronic kidney disease owing to medial calcification-induced incompressible infrapopliteal arteries at the ankle. In these patients, ABI is often elevated and falsely normalized despite the presence of lower extremity PAD. Therefore, sole reliance on the ABI value may lead to a missed diagnosis of lower-extremity PAD. In patients with normal ABI who are clinically suspected to have lower-extremity PAD, further diagnostic tests, including post-exercise ABI, toe-brachial index, pulse volume recordings, and duplex ultrasound should be performed to establish the diagnosis since a normal ABI does not definitely rule out a diagnosis of lower-extremity PAD [2, 3]. By using a volume plethysmograph, upstroke time at the ankle can be simultaneously and automatically calculated during the measurement of ABI without additional procedures [4]. Upstroke time at the ankle should be prolonged with hemodynamically significant stenosis or occlusion in the lower limb arteries. Since upstroke time may not be affected by the presence of calcified incompressible arteries, it is potentially helpful in the diagnosis of PAD in patients with normal ABI [5, 6].

The right ABI in patient 1 was 1.28 and was within the normal range, whereas the left ABI was 1.55 (> 1.40), which was indicative of incompressible left infrapopliteal arteries. In addition, right upstroke time was prolonged to 201 milliseconds (≥ 180 milliseconds) and was longer than that of the left ankle (130 milliseconds), indicating the possibility that the right ABI was falsely normalized owing to incompressible lower limb arteries at the ankle despite the presence of PAD in the right extremity.

In contrast to patient 1, both the right ABI (1.11) and the left ABI (1.03) were within the normal range and there was no difference in ABI between the left and right sides in patient 2. Therefore, it was more difficult to suspect the presence of lower-extremity PAD on the basis of the ABI values alone in patient 2 than in patient 1. However, bilateral upstroke times were prolonged to 206 milliseconds in the right ankle and 203 milliseconds in the left ankle, suggesting that bilateral ABIs were falsely normalized because of incompressible infrapopliteal arteries at the ankles despite the presence of PAD. In both patients, upstroke time was helpful for suspecting the presence of lower-extremity PAD in patients with normal ABI.

This case series indicates that ABI can be elevated and falsely normalized despite the presence of lower-extremity PAD in patients with incompressible infrapopliteal arteries, which are more prevalent in elderly patients with diabetes mellitus and/or advanced chronic kidney disease. In these patients, upstroke time at the ankle should be carefully checked to prevent a missed diagnosis of PAD or underestimation of cardiovascular risk in patients with normal ABI in addition to history-taking and vascular examination.

## Data Availability

Not applicable.

## Abbreviations

ABI: Ankle-brachial index
LAD: Left anterior descending coronary artery
PAD: Peripheral artery disease
